# Supplementary figures and images for: A phylogenetic analysis of Bromus (Poaceae: Pooideae: Bromeae) based on nuclear ribosomal and plastid data, with a focus on Bromus sect. Bromus
Source: PeerJ. 2022 Sep 28;10:e13884. doi: 10.7717/peerj.13884 (PMC9526414; doi:10.7717/peerj.13884)

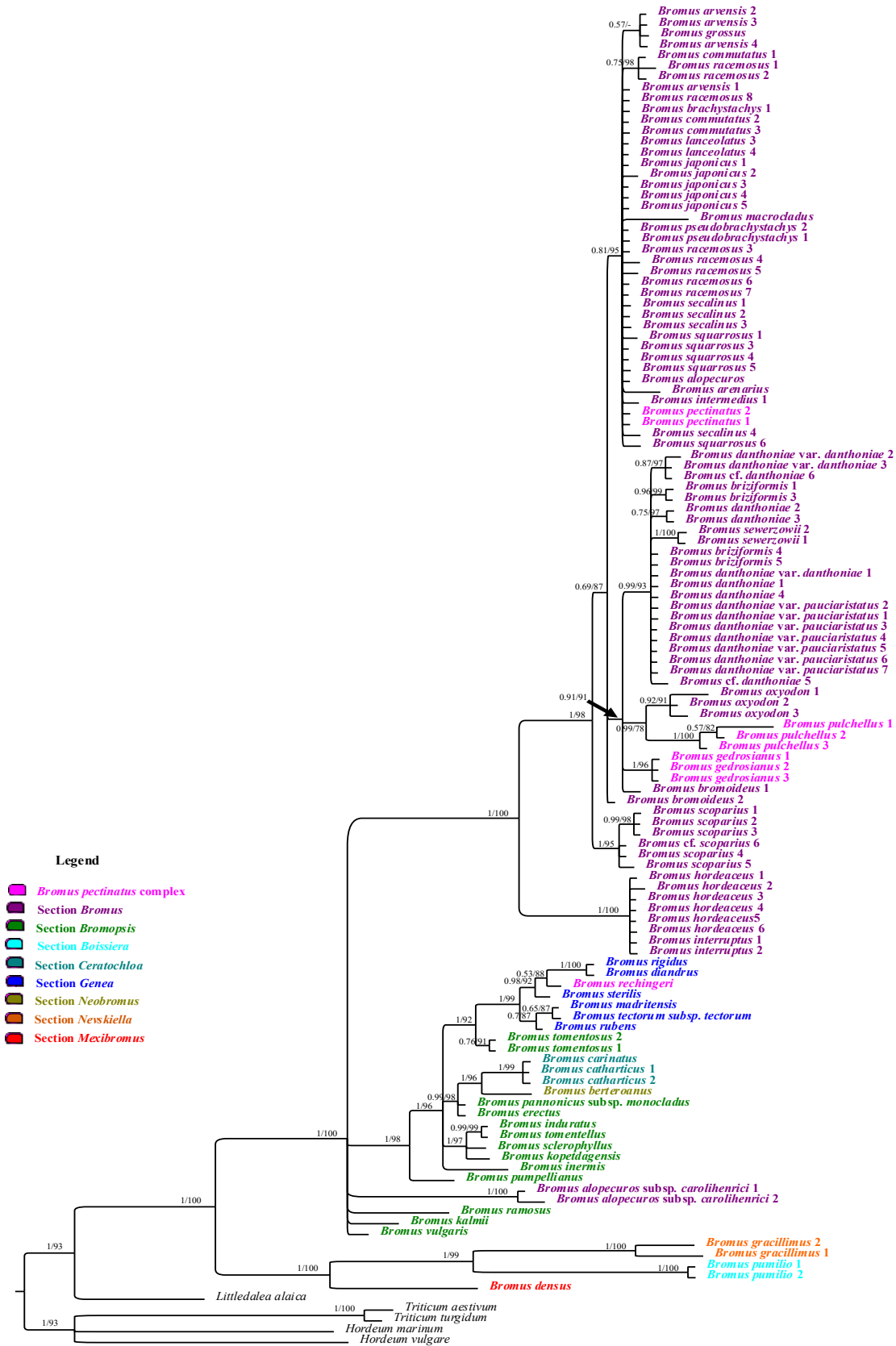

Supplement: Supplemental Information 5 — Bayesian posterior probabilities and maximum likelihood bootstrap support are indicated above the branches, respectively. Posterior probabilities <0.5 and bootstrap support <50% are indicated with a hyphen. [file peerj-10-13884-s005.pdf]

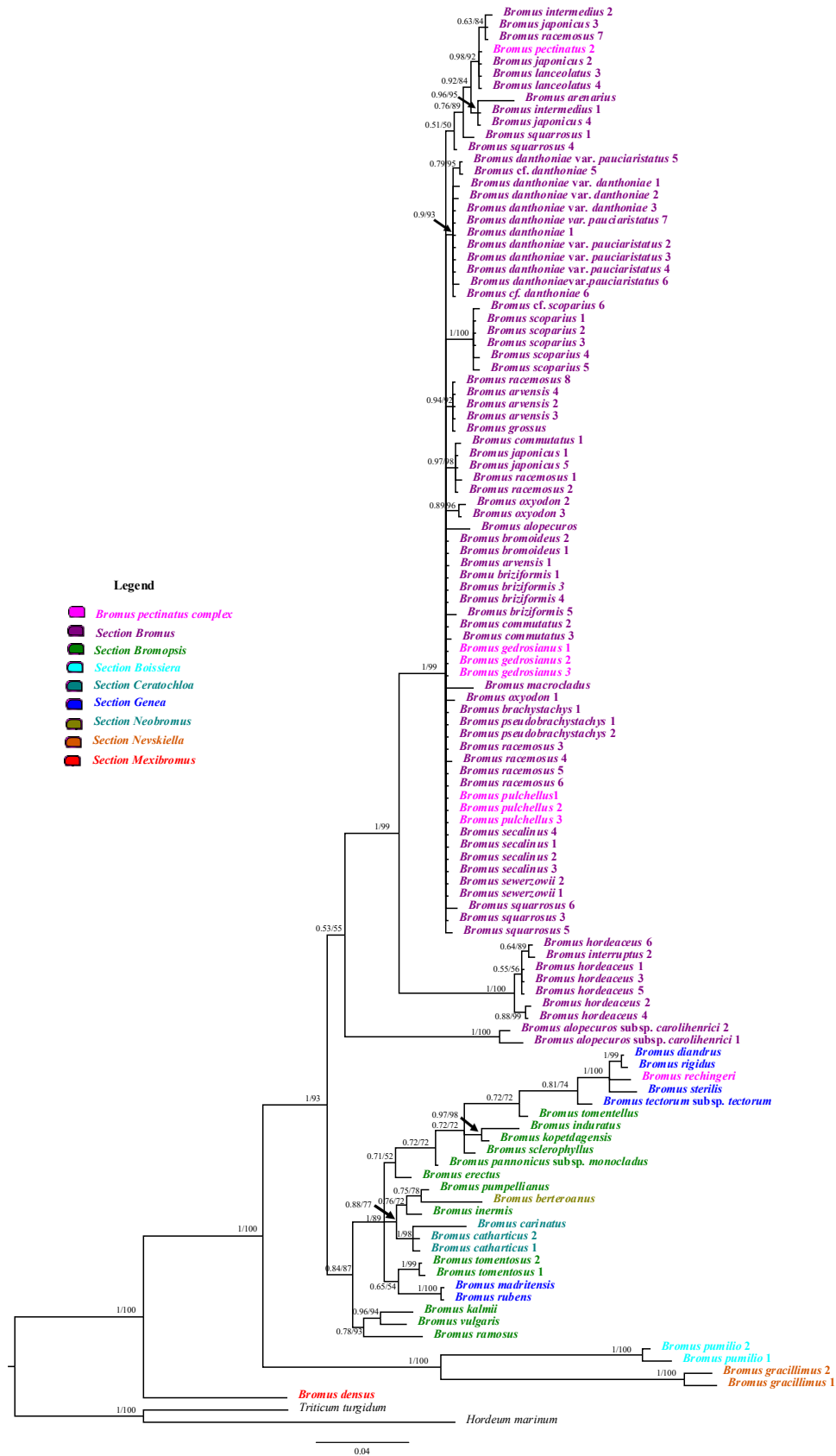

Supplement: Supplemental Information 6 — Bayesian posterior probabilities and maximum likelihood bootstrap support are indicated above the branches. [file peerj-10-13884-s006.pdf]

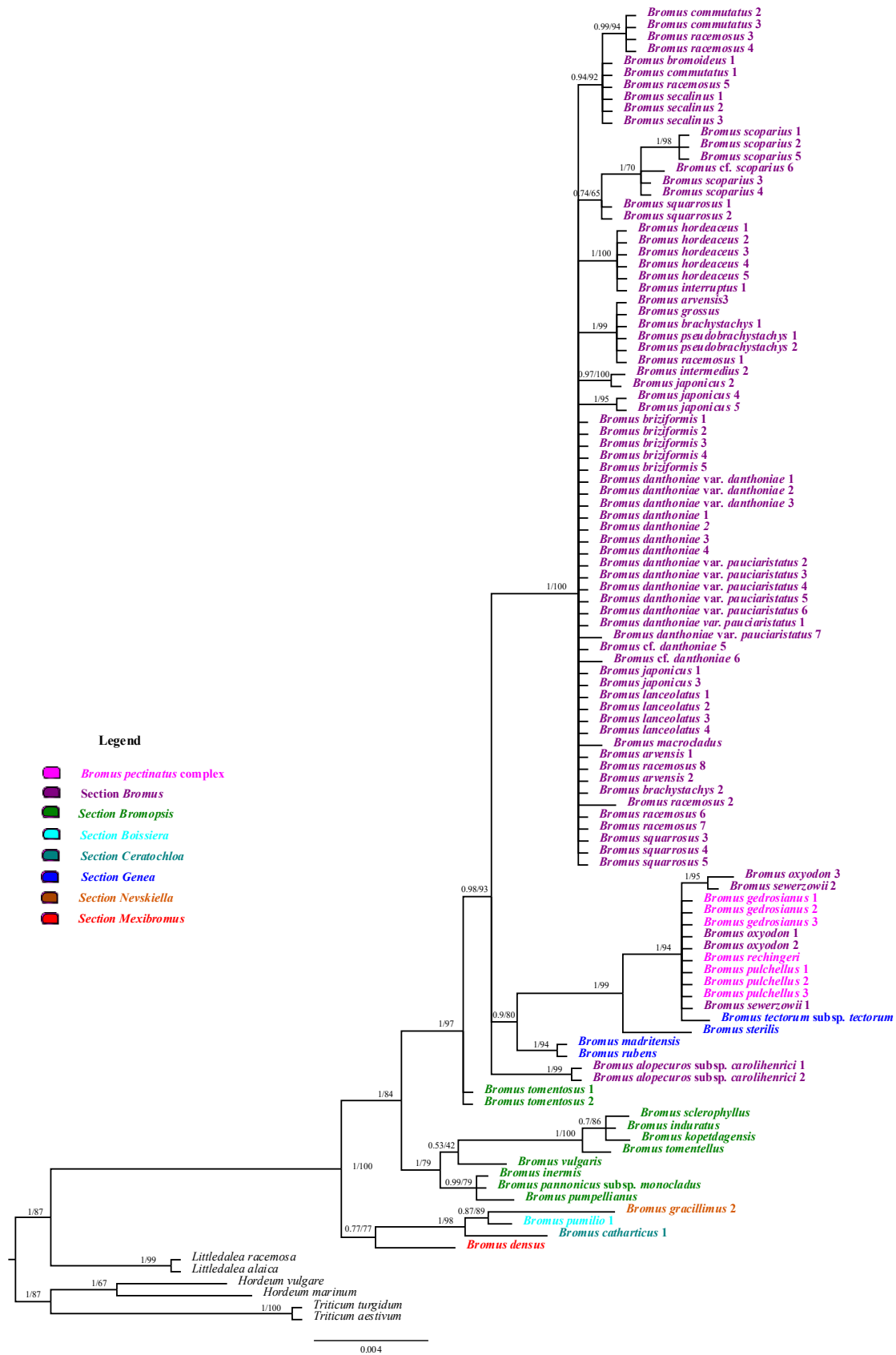

Supplement: Supplemental Information 7 — Bayesian posterior probabilities and maximum likelihood bootstrap support are indicated above the branches. [file peerj-10-13884-s007.pdf]

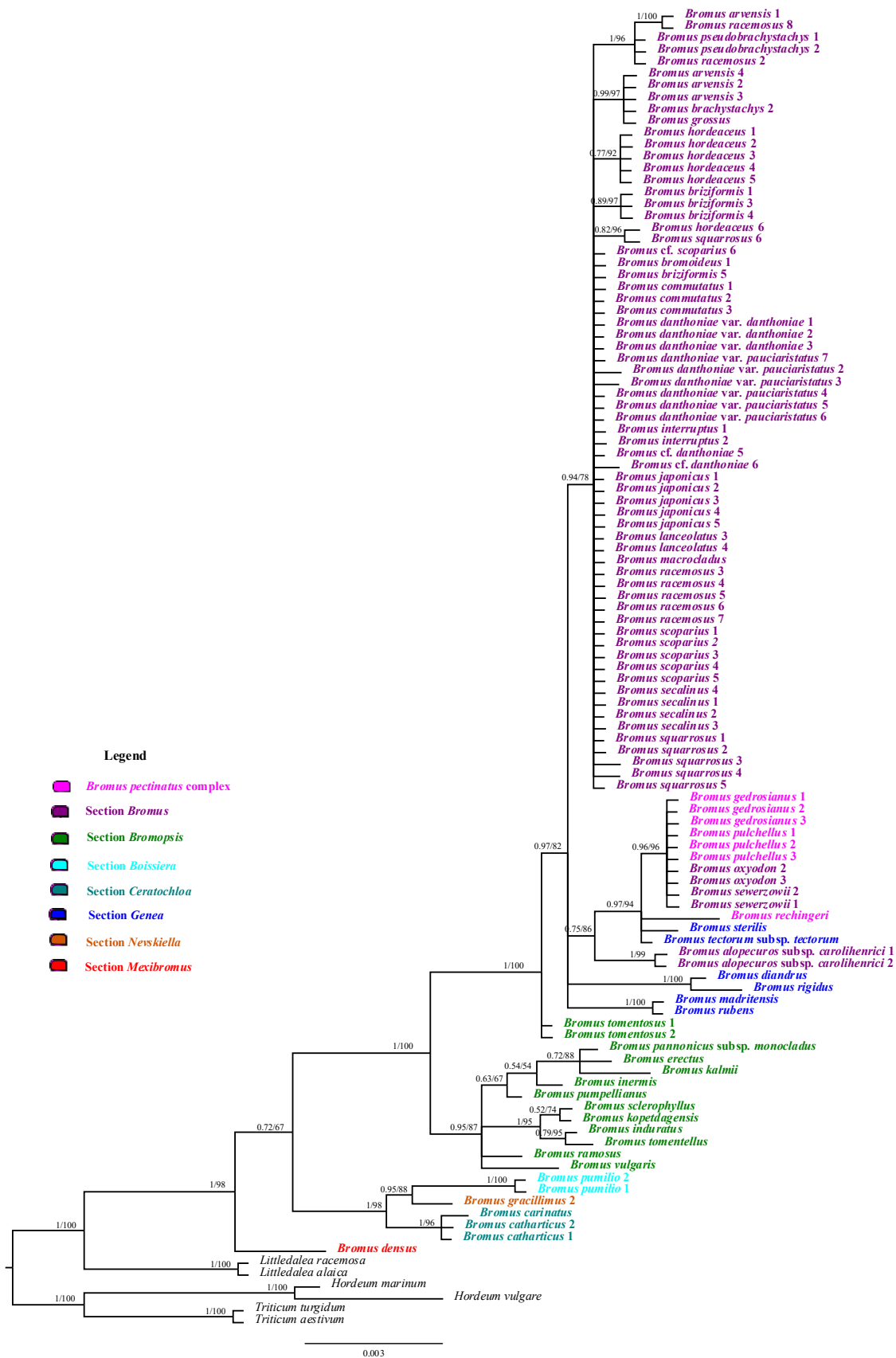

Supplement: Supplemental Information 8 — Bayesian posterior probabilities and maximum likelihood bootstrap support are indicated above the branches. [file peerj-10-13884-s008.pdf]

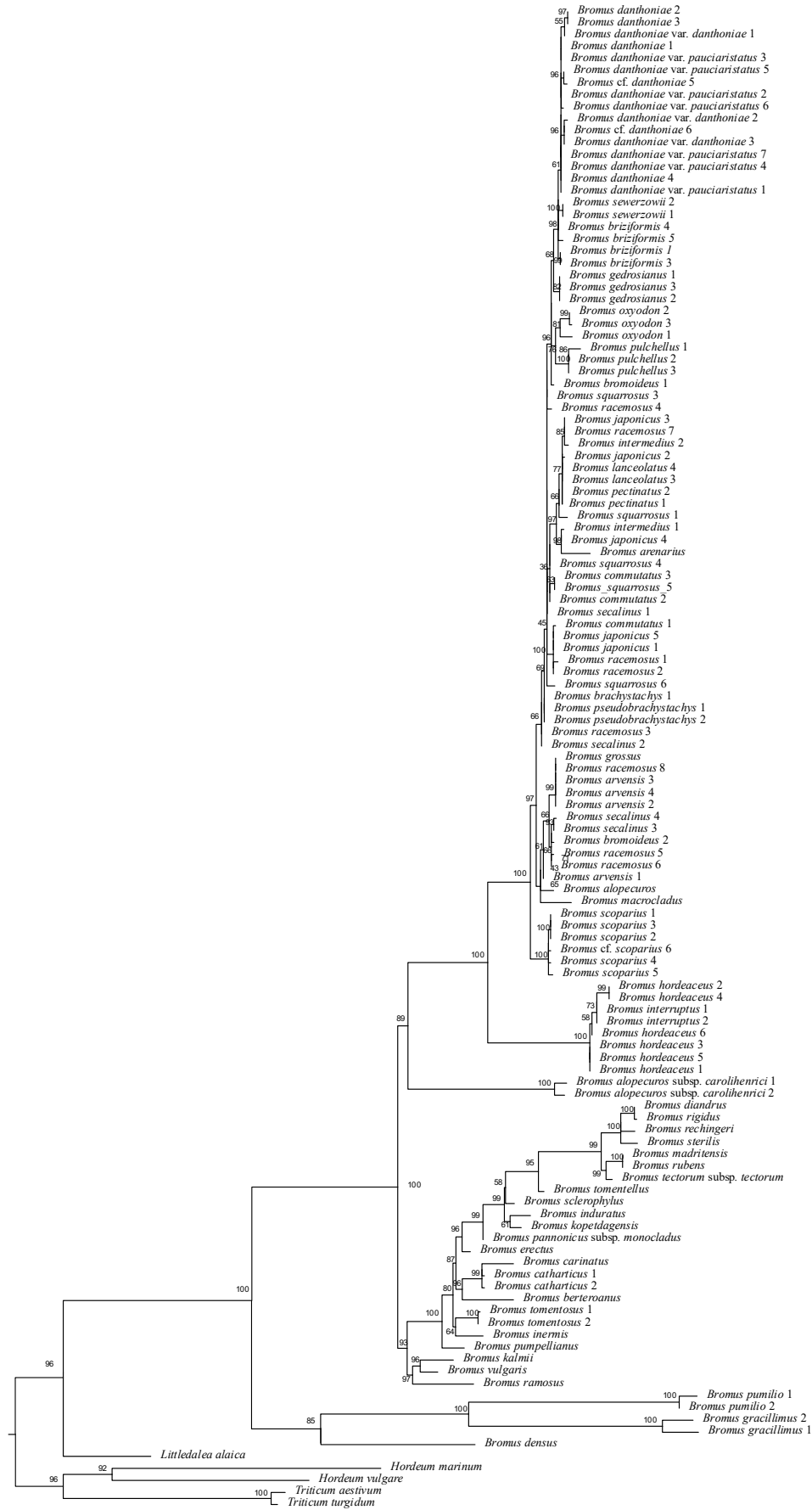

Supplement: Supplemental Information 9 — Numbers above branches are bootstrap values. [file peerj-10-13884-s009.pdf]

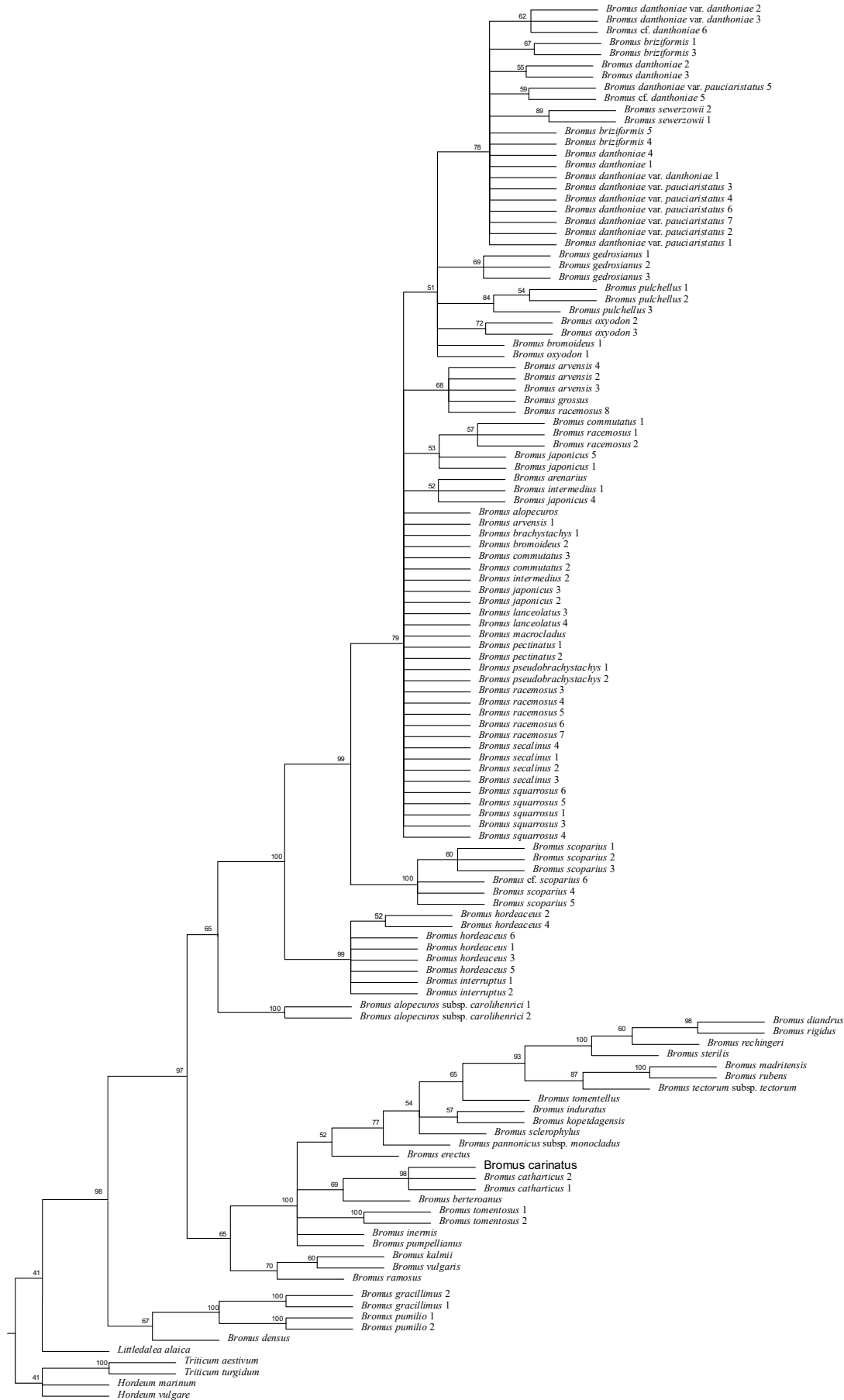

Supplement: Supplemental Information 10 — Numbers above branches are bootstrap values. [file peerj-10-13884-s010.pdf]

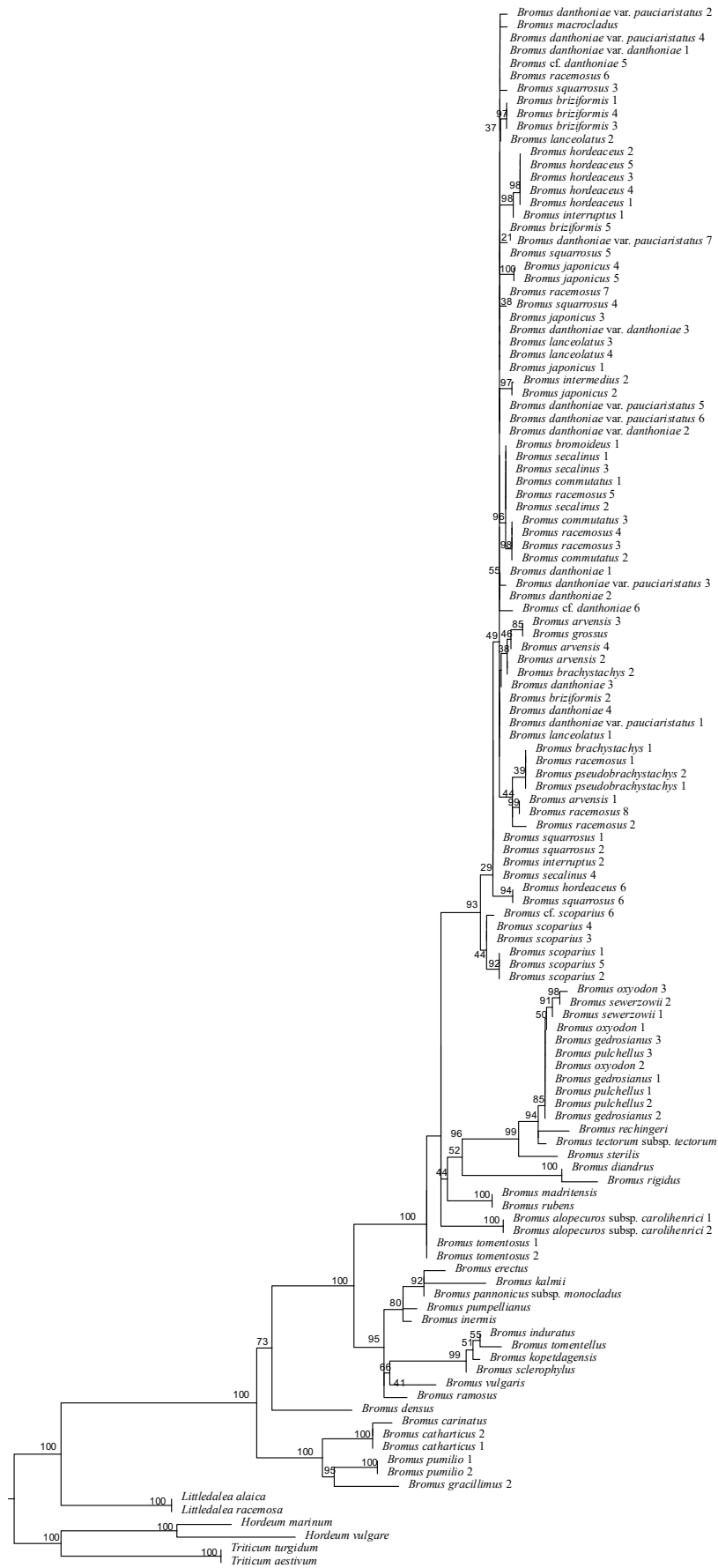

0.006

Supplement: Supplemental Information 11 — Numbers above branches are bootstrap values. [file peerj-10-13884-s011.pdf]

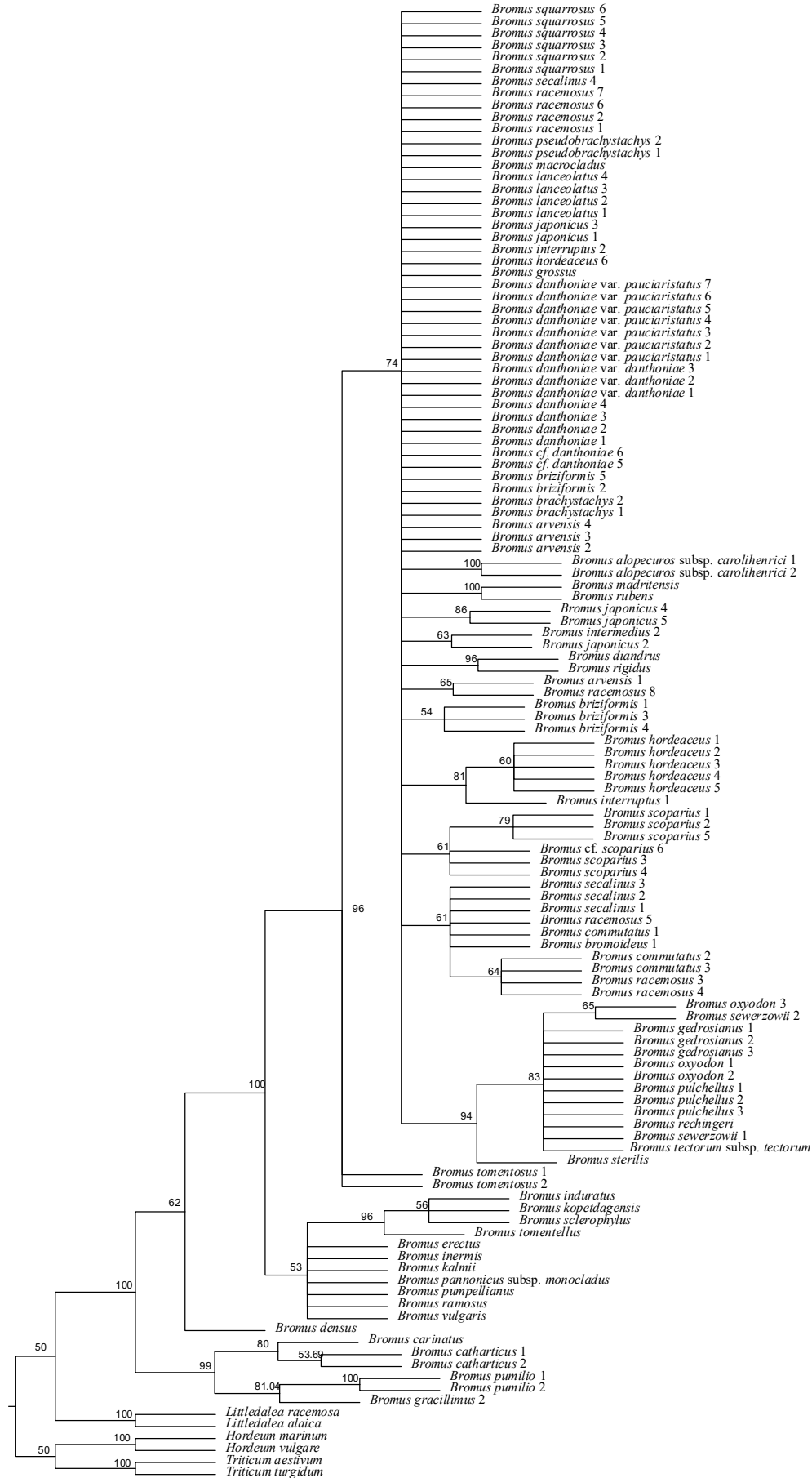

Supplement: Supplemental Information 12 — Numbers above branches are bootstrap values. [file peerj-10-13884-s012.pdf]
